# Excess Polθ functions in response to replicative stress in homologous recombination-proficient cancer cells

**DOI:** 10.1242/bio.018028

**Published:** 2016-09-09

**Authors:** T. Goullet de Rugy, M. Bashkurov, A. Datti, R. Betous, L. Guitton-Sert, C. Cazaux, D. Durocher, J. S. Hoffmann

**Affiliations:** 1 UMR1037, Le Centre de Recherches en Cancérologie de Toulouse (CRCT), 2 Avenue Hubert, Curien CS 53717, Toulouse 31037, Cedex 1, France; 2 UMR1037, CRCT, Université Toulouse, III-Paul Sabatier, Toulouse F-31000, France; 3 Equipe Labellisée Ligue Contre le Cancer, Toulouse F-31000, France; 4 The Lunenfeld-Tanenbaum Research Institute, Mount Sinai Hospital, 600 University Avenue, Toronto, Ontario, CanadaM5G 1X5; 5 Department of Agricultural, Food and Environmental Sciences, University of Perugia, Perugia 06121-06135, Italy

**Keywords:** Polθ, DNA polymerase theta, Synthetic lethality, High­-throughput screen

## Abstract

DNA polymerase theta (Polθ) is a specialized A-family DNA polymerase that functions in processes such as translesion synthesis (TLS), DNA double-strand break repair and DNA replication timing. Overexpression of *POLQ*, the gene encoding Polθ, is a prognostic marker for an adverse outcome in a wide range of human cancers. While increased Polθ dosage was recently suggested to promote survival of homologous recombination (HR)-deficient cancer cells, it remains unclear whether *POLQ* overexpression could be also beneficial to HR-proficient cancer cells. By performing a short interfering (si)RNA screen in which genes encoding druggable proteins were knocked down in Polθ-overexpressing cells as a means to uncover genetic vulnerabilities associated with *POLQ* overexpression, we could not identify genes that were essential for viability in Polθ-overexpressing cells in normal growth conditions. We also showed that, upon external DNA replication stress, Polθ expression promotes cell survival and limits genetic instability. Finally, we report that *POLQ* expression correlates with the expression of a set of HR genes in breast, lung and colorectal cancers. Collectively, our data suggest that Polθ upregulation, besides its importance for survival of HR-deficient cancer cells, may be crucial also for HR-proficient cells to better tolerate DNA replication stress, as part of a global gene deregulation response, including HR genes.

## Abstract (precis)

**Summary:** Our work suggests that Polθ upregulation may be crucial for homologous recombination (HR)-proficient cells to better tolerate DNA replication stress, as part of a global gene deregulation response, including HR genes.

## INTRODUCTION

The human genome contains 15 genes that encode DNA polymerases. Three of them, namely α, ε, and δ, have been extensively studied for their role in the error-free replication of the human genome. The twelve other ‘non-replicative’ or ‘specialized’ DNA polymerases have been mostly described as involved in mechanisms allowing the DNA to be repaired or replicated through DNA insults that block the progression of replicative polymerases, a process referred to as translesional synthesis (TLS). More recently, different studies including our own, supported the idea that the so-called ‘TLS’ polymerases may also be involved in other DNA-related events at the crossroad of DNA replication, repair and recombination (Bergoglio et al., 2013; Bétous et al., 2013; Fernandez-Vidal et al., 2014; and, for review, see Boyer et al., 2013; Hoffmann and Cazaux, 2010).

Within the TLS network, the DNA polymerase theta (Polθ) shows unique features, for example the existence of an N-terminal ATPase domain predicted to function as a DNA helicase, and a specific ability to function during DNA double-strand break repair (DSB) via a microhomology-mediated end joining (MMEJ) process (Kent et al., 2015; Mateos-Gomez et al., 2015; Zahn et al., 2015).

We and others have previously shown that Polθ is the most frequently overexpressed DNA polymerase in cancers and this overexpression is associated with an adverse clinical outcome. However, it is not yet clear whether Polθ overexpression is a bystander event occurring in aggressive tumour development or, more importantly, Polθ plays a driving role in tumour development and progression.

It has been recently proposed that high abundance of Polθ may result in an increased activity of the Polθ-mediated MMEJ pathway to compensate a defective homologous recombination (HR) repair, and might represent an adaptive mechanism favouring the survival of HR repair defective tumours such as approximately half of the epithelial ovarian cancers. Indeed, Polθ can mediate the repair of DSB through the error-prone alternative MMEJ DNA break repair pathway by inhibiting the recruitment of *RAD51*, an early step of HR (Ceccaldi et al., 2015; Newman et al., 2015). However, we recently reported that among the 101 breast tumours overexpressing *POLQ* analysed (Lemée et al., 2010), the majority did not present any alterations in HR. This prompted us to speculate here that *POLQ* overexpression might give a selective advantage of growth/proliferation also to HR-proficient tumours. We therefore explore in this work whether we could find genetic vulnerabilities associated with *POLQ* overexpression of HR-proficient cancer cells and if upregulation of Polθ could be selected during tumorigenesis in order to adapt to high levels of endogenous or external replicative stress. We also analysed whether *POLQ* deregulation in cancer could be part of a global deregulation of genes involved in the response of replicative stress *in vivo* by datamining gene expression data from published cancer studies. To the best of our knowledge this is the first study to demonstrate that *POLQ* overexpression confers a significant resistance to a general replication stress.

## RESULTS

### Lack of genetic robust vulnerabilities associated with *POLQ* overexpression in HR-proficient cells under normal growth conditions

**Fig. 1.**
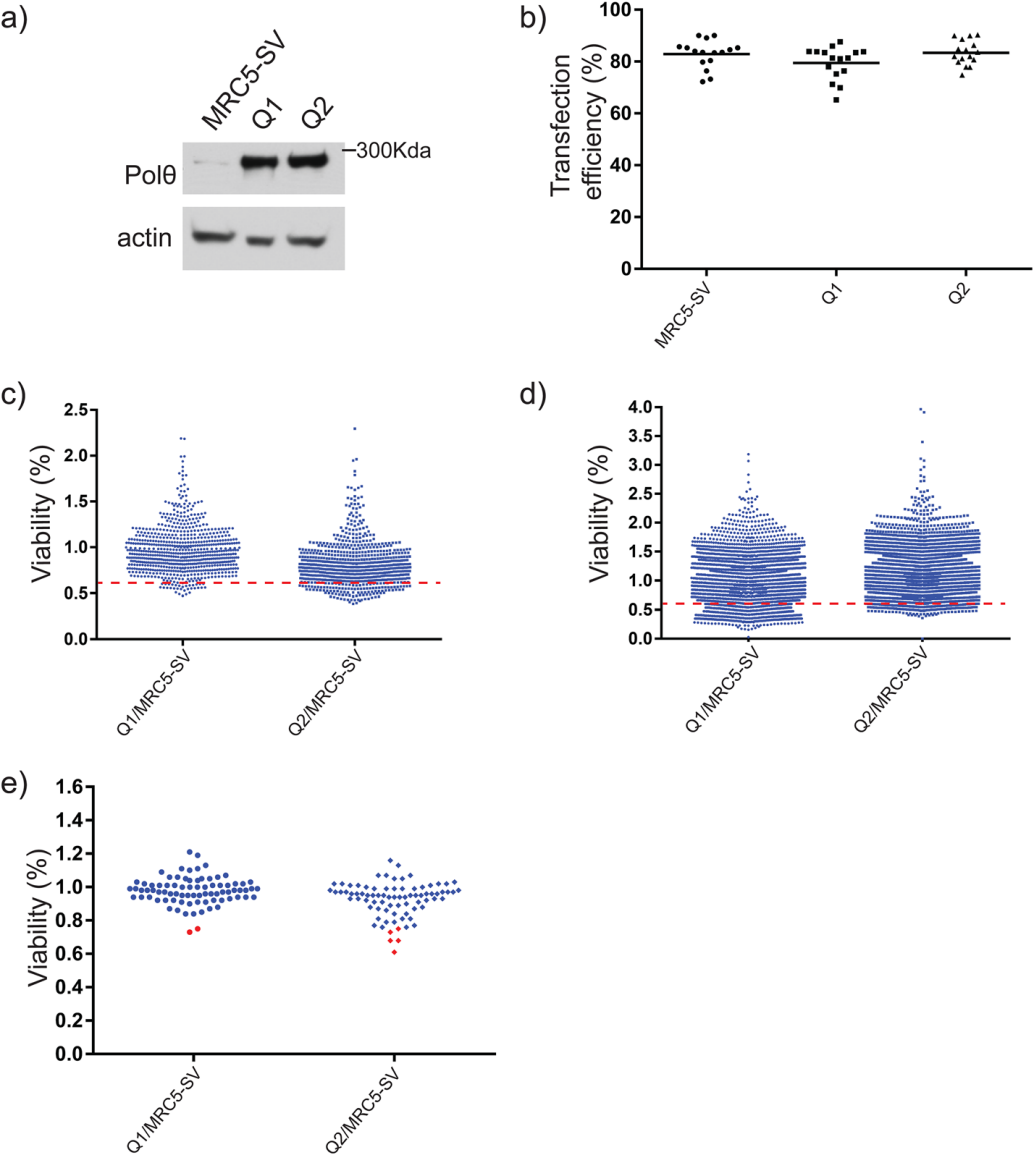
**siRNA-based high throughput screen.** (A) Analysis of Polθ protein levels in stably transfected clones and parental cell line. (B) Transfection efficiencies for plates from the primary screen. Transfection efficiency was calculated for each screened plate and for each of the cell line as follows: Efficiency=100−(average (siTOX)/average (mock)). Each dot represents the efficiency calculated for a 384 well plate of the screen. (C,D) Results obtained from kinome (C) and druggable (D) primary screens. siRNA from Dharmacon kinome and druggable library subsets were normalized to their negative siRNA control. Signals under the 0.6 threshold were considered potential synthetic lethal hits, and signals above as negative results. (E) Results from cherry-picked genes screened by MTS assay. The 80 best siRNA candidates obtained in the primary screen were retested in duplicate under similar conditions. Average of viability ratio compared to parental cell line for both experimental replicates is presented. SiRNA leading to a 20% decrease or more in the two clones as compared to MRC5 cells are highlighted in red.

In order to explore whether and how *POLQ* overexpression could be beneficial in HR-proficient cancer cells, we first investigated if we could find genetic vulnerabilities associated with *POLQ* overexpression of HR-proficient cancer cells under normal growth conditions. We carried out a siRNA screen in two MRC5-SV-clones overexpressing Polθ and compared it to their isogenic parental cell line. We established immortalized fibroblasts overexpressing Polθ (Fig. 1A) (Lemée et al., 2010) that were then transfected in 384-well format with siRNAs derived from the Dharmacon siGenome druggable and kinome libraries targeting a total of 5520 genes. After 72 h incubation following transfection, cells were fixed and stained with DAPI in order to identify nuclei and exclude dying cells. Plates were imaged with an automated microscope and viability was derived by counting cells in each well. The siTOX siRNA from Dharmacon, which efficiently kills cells, was used as a positive control. In each screen plate, the transfection efficiency was calculated as the percentage of cell death induced by siTOX as compared to mock condition. siRNA plates with a transfection efficiency below the 70% cut off were re-transfected until a transfection efficiency above 70% was achieved (Fig. 1B).

**Fig. 2.**
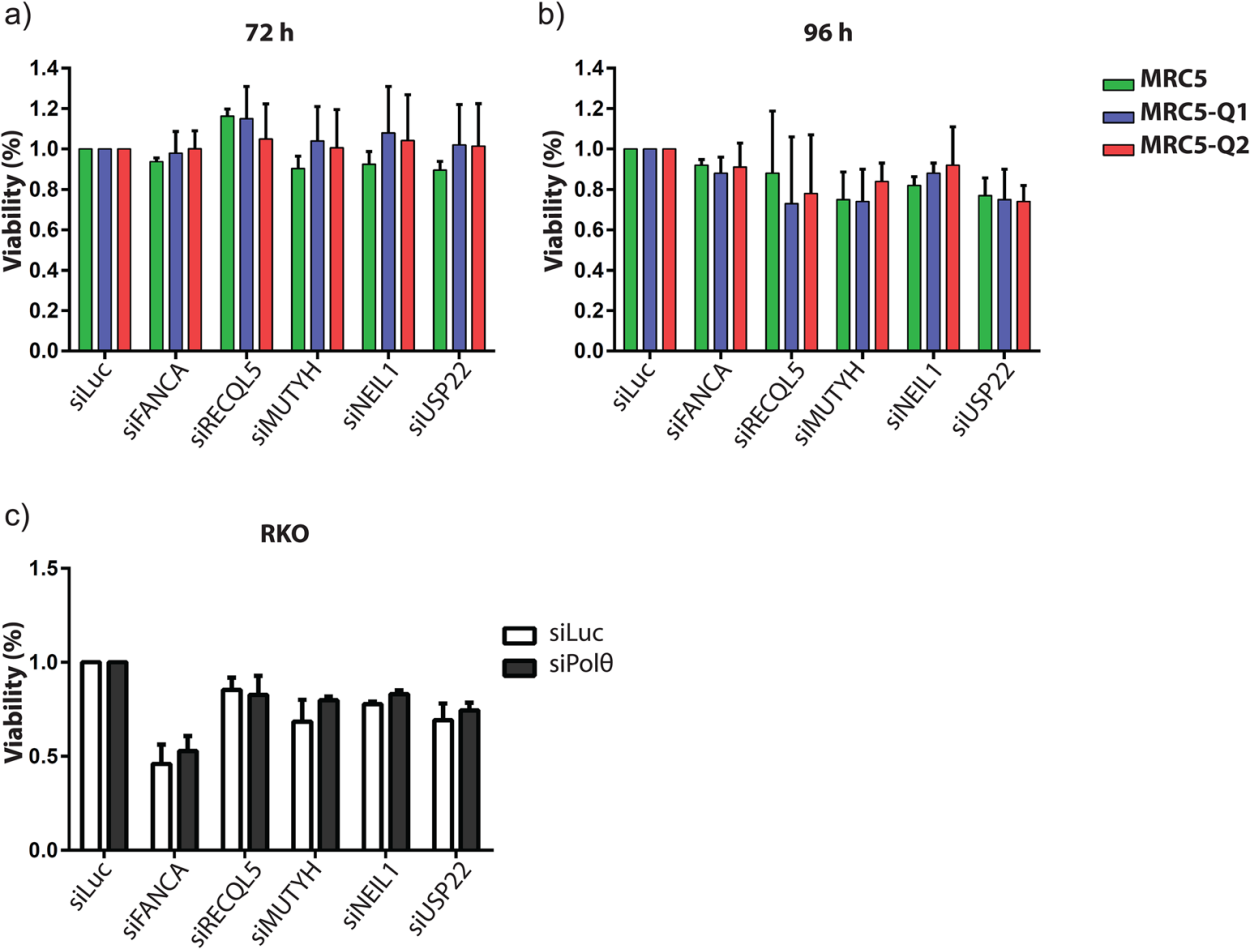
**Absence of synthetic lethality between Polθ and candidate genes.** (A,B) Potential hits were transfected in low throughput and cell viability was assessed by MTS test at 72 h (A) and 96 h (B) post-transfection. (C) RKO cells were transfected with siRNA against Polθ, and then seeded after 24 h incubation in a 96-well plate prior to transfection with the indicated siRNAs. Cell viability was assessed 96 h later by MTS test. Error bars represent standard deviation from three independent experiments.

To determine potential hit candidates in the primary screen, we calculated, for each siRNA pool, the ratio of cell viability of each overexpressing clone compared to that of the parental cell line (Fig. 1C,D). SiRNA pools that caused a cell survival ratio lower than 0.6 were considered as hits. Considering the high number of hits, we selected 80 candidates enriched in genes coding for proteins involved in DNA metabolism (about 1.5% of the genes tested in the primary screen). These candidates were then cherry-picked and retested for cell viability by the MTS assay (Fig. 1E; Fig. S1). The hits from this secondary screen (0.1% of primary screen) are presented in Fig. 2A. However, upon further validation, none of these siRNAs led to a significant decrease in viability of the *POLQ*-overexpressing cell lines (Fig. 2A). Since apoptosis in culture cells can take place 24 to 48 h following a genotoxic treatment, we extended the time frame of our assay, and monitored viability 96 h post transfection (Fig. 2B). While some siRNAs, under this condition, significantly increased cell death at 96 h post-transfection, we did not observe any significant difference between Polθ-overexpressing clones and the control cell line. Given that cancer cells are known to host disparate genetic rearrangements and rely on pathways involved in DNA checkpoints or repair (López-Contreras et al., 2012; Murga et al., 2011), we then decided to explore, by siRNA technology, the possibility of a functional relationship between up-regulated Polθ levels and the expression of specific genes within a tumorigenic phenotype. As a model, we chose the RKO colorectal cell line, which express high levels of endogenous Polθ, and knocked down Polθ prior to, 24 h later, a second knock-down round by the siRNA candidates previously selected as putative hits in fibroblasts (Fig. 2C). The cellular viability was then assessed 72 h later by the MTS assay. While some candidate genes induced enhanced cell death in a tumorigenic background compared to fibroblast (Fig. 2B,C), no statistically significant difference correlated with Polθ levels. We therefore failed to uncover strong synthetic lethal relationships between *POLQ* gene overexpression and genes coding for kinases and druggable enzymes under normal growth conditions.

### Polθ overexpression promotes cell survival in response to DNA replication stress

**Fig. 3.**
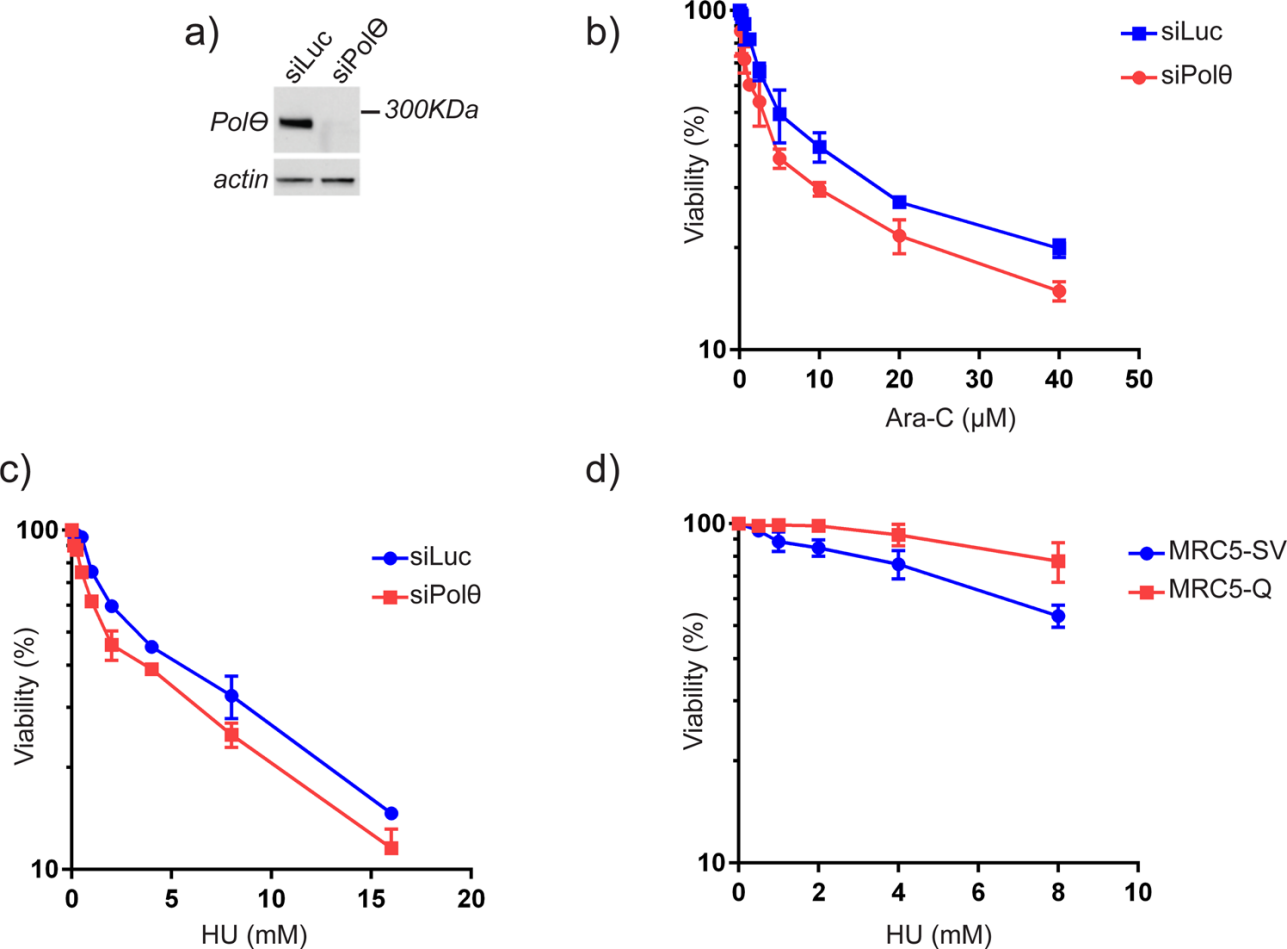
**Polθ is important for cell survival upon replicative stress.** (A) Validation of Polθ expression and knockdown in RKO cells. RKO were transfected with siRNA pools directed against Polθ and protein levels were estimated by western blot after 72 h. (B,C) Polθ deficiency leads to sensitisation of cancer cells to hydroxyurea (HU) (B) and Ara-C (C) treatments. RKO cells were transfected with siRNA targeting Luciferase and Polθ. On the following day, cells were treated with the indicated doses of drug for 4 h. Cell viability was assessed 48 h after treatment. (D) Polθ overexpression leads to resistance of cells to hydroxyurea treatment. MRC5-SV or MRC5-Q cells were treated with the indicated doses of drug for 4 h. Cell viability was assessed 48 h after treatment. Error bars represent standard deviation from three independent experiments.

Since *POLQ* over-expression might not provide a selective advantage for HR-proficient cells survival in the absence of external stress, we postulated that upregulation of Polθ could be selected during tumorigenesis in order to adapt to high levels of endogenous or external replicative stress, a condition that characterizes many cancers (Macheret and Halazonetis, 2015). We therefore compared cell survival of mock- and Polθ-depleted cells following treatment with replication stress-inducing agents. Thus, we treated RKO cells, that naturally overexpress Polθ, with hydroxyurea (HU) and cytarabine (Ara-C), two drugs that inhibit the DNA replication fork progression by depleting the nucleotide pool or chain termination, respectively (Galmarini et al., 2001; Krakoff et al.). Our results show that Polθ depletion by siRNA (Fig. 3A) reduced cell survival, as monitored by the MTS viability test, in response to Ara-C and HU (Fig. 3B,C). Also, we performed the mirror experiment by monitoring the survival of Polθ-overexpressing MRC5 cells upon HU treatment. We found that high abundance of Polθ increased cell survival, as compared to the control isogenic cells (Fig. 3D), confirming that the abundance of Polθ can modulate resistance to replicative stress.

**Fig. 4.**
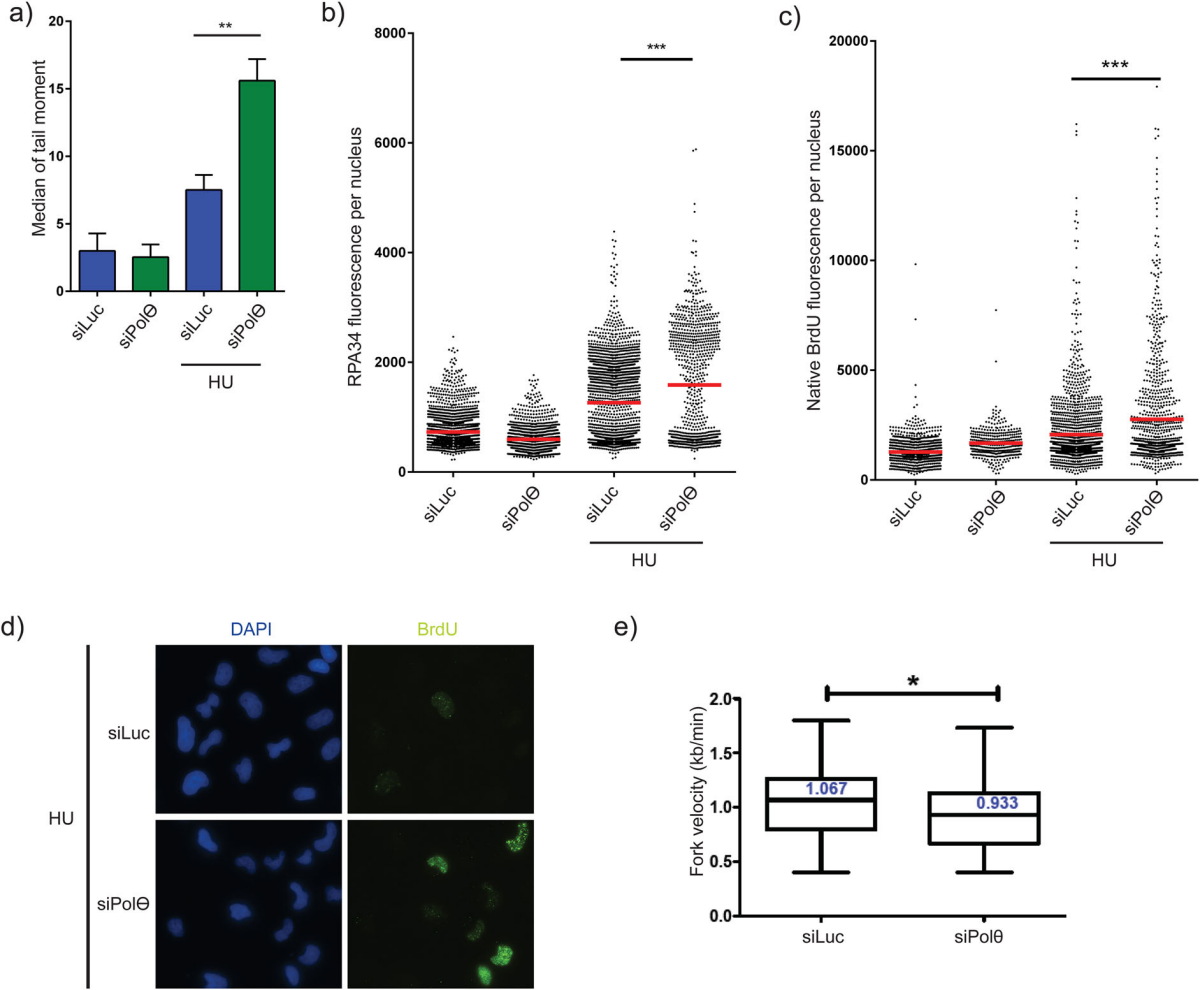
**Polθ prevents genomic instability induced by replicative stress.** (A) Replication­-induced DNA DSBs in Polθ-depleted cells after treatment with hydroxyurea (HU). RKO cells were treated with 4 mM of hydroxyurea prior to resuspension in low-melting agarose and electrophoresis required to perform the neutral comet assay. At least 100 nuclei were quantified per condition. A two-tailed *t*-test was used to assess statistical significance, **P<0.01. The graph shows the mean±s.d. from three independent experiments. (B,C) Increased ssDNA in nuclei from Polθ depleted cells. (B) RKO were treated with indicated doses of hydroxyurea 48 h after transfection cells with control siRNA (siLuc) or siRNAs targeting Polθ. RPA34 intensity was detected in nuclei after nuclear pre-extraction (a minimum of 900 nuclei per condition were quantified). (C) Transfected MRC5-SV were cultivated during 36 h with BrdU in culture medium before treatment with HU. BrdU was detected by immunofluorescence microscopy (a minimum of 350 nuclei per condition were quantified). Two tailed Mann–Whitney tests were performed to assess statistical relevance of populations (***P<0.001). Results of two independent experiments are shown. (D) Representative images of the cells analysed in B,C. (E) Polθ depletion influences fork velocity. Twenty-four hours after transfection of the RKO cell line with control (siLuc) or Polθ siRNA, RKO cells were labelled successively with 50 µM IdU (Sigma-Aldrich) for 15 min, 100 µM CldU for 15 min and 3 h 200 mM thymidine. DNA combing was performed as described previously (Fernandez-Vidal et al., 2014). The number of bi-colour forks analysed in this experiment are 74 and 64 for siLuc and siPOLQ conditions, respectively.

In order to better understand the mechanism by which Polθ-depleted cells can be sensitized to DNA replication stress, we used a neutral comet assay to analyse formation of HU-induced DSBs (Fig. 4A). Quantification of comet tail moments revealed that Polθ loss causes higher levels of DSBs following HU treatment, suggesting that Polθ prevents DSB formation in response to DNA replication stress. In addition, we monitored accumulation of ssDNA by detecting ssDNA-bound protein RPA by immunofluorescence after HU treatment. We found that the percentage of cells with strong RPA signal significantly increased following Polθ knockdown (Fig. 4B). To confirm this result, immortalized cells were grown in a culture medium containing the BrdU nucleotide analogue (BrdU) to detect ssDNA formation through a native BrdU labelling assay (Raderschall et al., 1999). This assay enables the visualization of exposed ssDNA, notably following uncoupling of DNA replication forks and the replicative helicase. We observed that a significant increase of cells positive for BrdU staining was detected in the absence of Polθ upon HU treatment (Fig. 4C,D). Collectively, these findings demonstrating an accumulation of ssDNA when Polθ is knocked down, further support a role of Polθ in the response to replicative stress.

Finally, we analysed the DNA replication forks *in cellulo* both at the whole genome and at the single molecule levels by performing a DNA fibre combing technique, a method for labelling tracts of new DNA synthesis *in vivo* enabling us to monitor replication fork progression as nascent DNA at the level of individual replicating DNA molecules. This technique relies on two consecutive incorporations of different halogenated nucleotides which can label two subsequent periods of DNA synthesis. The DNA molecules that have incorporated these analogues can be visualized by fluorescence microscopy and DNA track lengths can be quantified (see the Materials and Methods section). We found a mild but significant reduction of the replication track length in Polθ-depleted cells compared to control cells (Fig. 4E), suggesting that the role of Polθ in the response to replicative stress may occur in unstressed cells directly at natural replication barriers.

### *POLQ* overexpression strongly correlates with HR gene expression in cancer

**Fig. 5.**
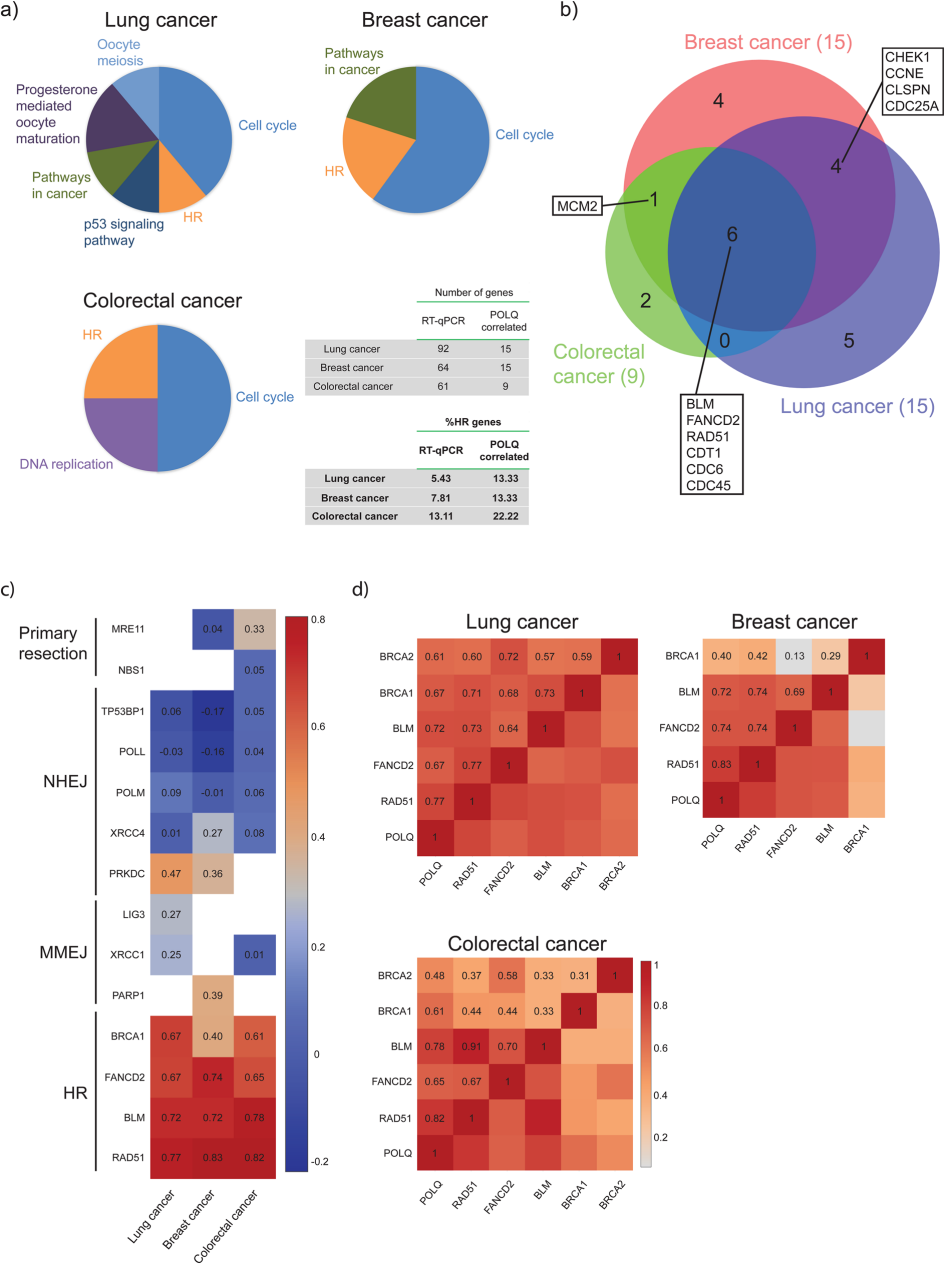
**Polθ expression positively correlates with HR but not MMEJ genes in solid tumours.** (A) A Pearson’s test was used to compare *POLQ* mRNA expression and expression of any other analysed gene within each cohort. The number of patients analysed for lung, breast and colorectal cancer was 103, 221, and 52, respectively (Lemée et al., 2010; Pillaire et al., 2010; Allera-Moreau et al., 2012). A coefficient superior to 0.65 was considered associated to a positive correlation. Pathway enrichment analysis was performed using the KEGG pathway database. Pie charts report the pathways detected by KEGG analysis in relation to the number of genes in each pathway. (B) Venn diagram representing different correlating genes shared by the different tumour types. (C) Heatmap of the Pearson coefficients for correlation of expression between *POLQ* and genes involved in different DSB repair pathways. A white block is indicative of unavailable data. (D) Heatmap showing correlation between different genes involved in HR. A more intense colour indicates a stronger positive correlation.

To explore whether *POLQ* deregulation in cancer could be part of a global deregulation of genes involved in the response of replicative stress, we thought to go back to our previously reported real-time PCR data related to DNA samples from breast, colorectal or lung cancer patients (Allera-Moreau et al., 2012; Lemée et al., 2010; Pillaire et al., 2010), and address whether the expression of *POLQ* was correlated with the expression of other genes. By using Pearson coefficient of correlation with a threshold of *P*=0.65, we searched for genes that significantly correlated with *POLQ* expression. Indeed, the Pearson coefficient is a statistical tool indicating the strength of a linear correlation; a Pearson coefficient of 0 being the absence of correlation and a coefficient of 1 a perfect positive correlation. Interestingly, we found that *POLQ* expression in breast, colorectal and lung cancers is positively correlated with the expression of 15 out of 64, 9 out of 61 and 15 out of 92 genes, respectively (Fig. 5A,B; Fig. S2). Next, we investigated, by KEGG mapping, the molecular pathways relevant to such correlated genes (Kanehisa et al., 2014). KEGG (Kyoto Encyclopedia of Genes and Genomes) is a bioinformatics resource allowing quantification, from a list of genes, of the potential enrichment in some biological processes and pathways. Notably, our analysis consistently revealed gene networks associated with the cell cycle and homologous recombination in the three cancers studied. Indeed, 2 out of 15, 4 out of 9 and 2 out of 15 correlated genes were identified as HR genes in 103 lung, 52 colorectal and 221 breast tumour samples, respectively. This analysis showed an enrichment of the HR pathway in genes positively correlating with *POLQ* expression (*P*-values of hypergeometric test: 1.25×10^−5^, 1.46×10^−5^ and 1.25×10^−5^). While it is possible that the original real-time PCRs, performed on a set of genes involved in DNA repair and DNA replication, may arguably have led to biased conclusions, further data analysis revealed that the percentage of HR-related genes that correlated with *POLQ* overexpression was consistently higher than in the gene population subject to PCR analysis. Moreover, the expression of *FANCD2*, an HR gene not referenced in the KEGG ‘HR’ pathway, also correlates with *POLQ* (Fig. 5B). Furthermore, six genes correlate with *POLQ* expression in the three different cancer types. Of these genes, three of them have previously been described in the literature shown to be involved in HR mechanism (*BLM*, *FANCD2* and *RAD51*), while the others (*CDT1*, *CDC6* and *CDC45*) are well-described regulators of DNA replication origins, a molecular pathway in which we recently implicated Polθ (Fernandez-Vidal et al., 2014).

Although Polθ was recently described as a key factor of MMEJ (Kent et al., 2015; Mateos-Gomez et al., 2015), *POLQ* expression did not correlate with the expression of several genes encoding for core MMEJ factors (*PARP1*, *XRCC1* and *LIG3*) (Fig. 5C). *POLQ* expression did not correlate either with the expression of genes involved in NHEJ or with genes involved in DNA end resection, a step shared by MMEJ and HR (coefficient<0.5) (Truong et al., 2013). Next, we calculated a Pearson coefficient for each pair of genes involved in HR and *POLQ*. Strikingly, HR genes expression strongly correlated with each other with the exception of *BRCA1* in breast and colorectal cancer and *BRCA2* in colorectal cancer (Fig. 5D).

Importantly, the three HR genes whose expression most strongly correlated with *POLQ* expression (Fig. 5B), namely *RAD51*, *FANCD2* and *BLM*, have been shown to be major actors in response to replicative stress (Chaudhury et al., 2013; Chen et al., 2016; Zellweger et al., 2015). Therefore, these data collectively support the notion that *POLQ* overexpression is part of a global gene expression reprogramming that specifically integrates HR genes to respond to endogenous and/or external-induced replication stress.

## DISCUSSION

We and others have demonstrated that Polθ expression is a strong prognostic factor in different types of cancers (Allera-Moreau et al., 2012; Lemée et al., 2010; Pillaire et al., 2010). Recent studies have demonstrated the important role of Polθ in DNA repair under stress conditions and suggest that Polθ overexpression may play a key role in mechanisms of protection against genotoxic therapies (Yousefzadeh et al., 2014), especially when other repair pathways are no longer functional (Ceccaldi et al., 2015; Mateos-Gomez et al., 2015). Indeed, in a HR-deficient background, Polθ becomes essential for cancer cells by promoting MMEJ and preventing toxic RAD51 structures. However, the frequency of Polθ overexpression, especially in sporadic breast cancer, suggests that Polθ is not only involved in a compensatory mechanism that rescues HR deficiency, but is likely to provide HR-proficient tumours with a growth advantage. Indeed, while the proportion of HR-proficiency in sporadic cancers is difficult to quantify, somatic mutations in *BRCA* genes in sporadic breast cancer is estimated to be around 10% (Futreal et al., 1994; De Leeneer et al., 2012). To obtain new insights regarding the contextual relevance of Polθ overexpression in tumours, and identify possible mechanisms associated with its mode-of-action, we initially studied the correlation between the expression of *POLQ* and that of the genes involved either in DNA repair or replication. Surprisingly, the expression of genes coding for MMEJ factors did not correlate with *POLQ* expression. However, the HR pathway was significantly enriched in genes that were correlated with *POLQ*. In this regard, *BLM*, *FANCD2* and *RAD51* show a very strong correlation that, interestingly, was already reported for *FANCD2* and *RAD51* in ovarian cancer (Ceccaldi et al., 2015). However, in our dataset, *BRCA1/2* genes show a weaker correlation with Polθ expression in lung cancer and no correlation in colorectal and breast cancer. *MRE11* and *NBS1*, two genes coding for proteins of the MRN complex involved in primary resection, an early step in the HR pathway, do not instead correlate with Polθ expression. Notably, published work has repeatedly suggested that a number of proteins involved in the HR pathway play a role in the management of stalled replication fork under replicative stress (Carr and Lambert, 2013). Moreover, *RAD51* and *BLM* have been described to be involved in remodelling stalled replication fork in a mechanism described as ‘fork reversal’ (Neelsen and Lopes, 2015). These observations prompted us to evaluate the role of Polθ in the response to chemotherapeutic agent that mediates replicative stress. We used Ara-C, a drug currently used in clinics, and HU to treat cells depleted for Polθ. Interestingly, our findings show that Polθ depletion sensitizes cells to both agents. This phenotype was accompanied with an increase of DNA DSB and an increase in ssDNA upon HU treatment (Fig. 4A-C). In this regard, one possible explanation could be that the absence of Polθ decreases fork restarts and increases fork stalling or fork collapse. The fact that Polθ is frequently overexpressed in a wide range of malignant tumours highlight the importance to design new therapeutic strategies. One possibility might be to target Polθ and thus, resensitize cancer cells to chemotherapeutic agents that induce replicative stress. However, while this strategy may appear attractive, it must be noted that Polθ contains several catalytic domains that may both contribute to chemo resistance, thereby ruling out conventional drug discovery rationales based on the development of enzymatic inhibitors (Li et al., 2011). Another strategy would be to target a cognate pathway that is engaged within the dynamic context of Polθ overexpression. This strategy was explored in the past by targeting kinases involved in mitotic processes in cells with endogenous replicative stress (Luo et al., 2009). In this regard, we attempted to unveil potential targets by means of a systematic siRNA-based screen using cells that overexpressed Polθ and, in parallel, cells displaying basal levels of polymerase expression. Despite good transfection efficiencies and a high dynamic range (i.e. positive control siTOX induces at least 80% cell mortality), our attempt at targeting 5550 different genes did not reveal any statistically significant hits and, therefore, any evidence for synthetic lethal relationships between excess Polθ and a given metabolic pathway in unstressed cells. This, in turn, supports the notion that there is no overt metabolic dependency in Polθ overexpressing cells in these conditions. Recent studies have demonstrated that Polθ is a bona fide MMEJ protein and revealed insights concerning its biological roles toward DNA repair in cancer cells. However, taken together, our results support a potential role of this protein in DNA replication stress response, which may also be essential in an HR-deficient background. The role of Polθ in response to replicative stress could therefore represent a pharmacological target in cancer sensitization to chemotherapy in both HR-proficient and HR-deficient tumours.

## MATERIALS AND METHODS

### Cell lines and plasmid

MRC5 is the parental cell line from lung fibroblastic origin and is immortalized by SV40. MRC5-Q1 and Q2 were transfected with a plasmid coding for human Polθ labelled with Flag tag, and overexpressing clones were selected as previously described (Lemée et al., 2010) using hygromycin B as selective pressure. RKO were obtained from ATCC (CRL­2577™) and MRC5-SV parental cell line from ECACC (MRC5-SV2, catalogue number: 84100401).

### siRNA and transfection reaction

The ‘kinome’ (*n*=720) and ‘druggable’ (*n*=4440) siRNA subsets used for this study were obtained from the human, SMARTpool library from Dharmacon. siRNAs were transfected to yield a final concentration of 40 nM. For transfection, 10^3^ MRC5 cells were seeded in 384-well plates. After 24 h, transfection reactions were performed using MRC5 cell Avalanche reagent (EZbiosystem) in antibiotic-free medium. After 6 h of incubation, the medium was changed to reintroduce antibiotic and selective pressure for Polθ overexpression.

### Screening

Following a 72 h incubation with siRNAs, cells were fixed using fresh 2% PFA for 15 min and then permeabilized with Triton X-100 0.1% for 30 min. Cells were then incubated with DAPI 5 mg/ml for 15 min and washed with PBS. High­-capacity acquisition of fluorescent cells nuclei images was obtained with an InCell analyser 6000 (GE Healthcare) with a ×20 objective lens. Image analyses were carried out with the Columbus software (Perkin Elmer).

### MTS viability assay

Cells were reverse-transfected in triplicate in 96-well plates with siRNA targeting candidate genes. Medium was changed 6 h post-transfection and hygromycin B was added to restore selection pressure when needed. MTS was added 72 h or 96 h after transfection of cells and incubated 3 h at 37°C. Viability was measured using a spectrophotometer at 590 nm.

### Neutral comet assay

Cells were mixed with low melting agarose and then spread on microscopic slides (Kit ref. 4250-050-K, Trevigen). Slides were brought to 4°C for about 10 min to allow solidification of the gel. Samples were lysed for 1 h at 4°C and then rinsed in electrophoresis buffer for 30 min. Electrophoresis was then run at 20 V for about 50 min at 4°C, after which slides were incubated in precipitation buffer at room temperature for 35 min, rinsed in 70% ethanol under agitation and dried at 37°C for 15 min before incubation with SYBR^®^Gold. Finally, slides were dried again before imaging.

### Immunofluorescence

Cells were grown on glass coverslips and pre-extracted with NP-40-based buffer for 15 min [20 mM HEPES pH 7.4, 0.5% NP40, 20 mM NaCl, 5 mM MgCl2, 1 mM DTT and 1× Halt TM protease/phosphatase inhibitors (ThermoFisher Scientific)] followed by fixation with 4% paraformaldehyde (PFA) incubated for 15 min at RT. After fixation, cells were washed in PBS and blocked with 5% BSA (Euromedex) in PBS. Cells were incubated overnight at 4°C with primary antibodies against BrdU (BD bioscience 347583) or RPA34 (Clabiochem NA18) in PBS (1:100 and 1:200, respectively). Then coverslips were washed with PBS, and then incubated with Alexa Fluor 488 or 555 goat anti-mouse or anti-rabbit (1:1000; Molecular Probes) for 1 h at RT in PBS. DNA was counterstained with DAPI and coverslips mounted on microscopy-slides using ProLong Diamond (Thermo Fisher).

### DNA combing

Cells were successively labelled for 15 min with 50 µM IdU (Sigma-Aldrich) and 100 µM CldU (ICN) and incubated 3 h with 200 mM thymidine. Genomic DNA was prepared in agarose plugs (0.5×10^5^ cells/plug), and DNA combing was performed as described previously (Fernandez-Vidal et al., 2014). IdU and CldU were detected with monoclonal mouse (347580, 1:20, Becton Dickinson) and rat anti-BrdU antibodies (Abc117–7513, 1:20, Seralab), respectively. Signals were captured with a CoolSNAP HQ camera (Photometrics) on a Leica DM IRB equipped with a 63×/1.4 PL APO objective. Measurements were performed with MetaMorph (Universal Imaging Corp.). Fork speed was calculated by dividing the median track size by the labelling time and the non-parametric test of Mann–Whitney was used to data sets from different cell lines.

### Statistical and bioinformatics analysis of gene expression from tumour cohorts

Written informed consent was obtained from all patients before testing. All informed consents were collected and stored in the Pathology Department. This study was approved by the Ethics of Human Research Committee at the Pathology Toulouse Hospital. The experiments conformed to the principles set out in the WMA Declaration of Helsinki. RT-qPCR data from human tumour samples were retrieved from 221 breast, 94 NSCLC and 52 colorectal cancer samples previously (Allera-Moreau et al., 2012; Lemée et al., 2010; Pillaire et al., 2010). Correlation with *POLQ* gene expression was assessed using Pearson test with a *P*=0.65 cut-off. For each cancer type, total gene set and *POLQ*-correlating genes were analysed for pathway enrichment using the KEGG database (Kanehisa et al., 2014). Co-occurrence of correlation among different tumour types was assessed using Venny (http://bioinfogp.cnb.csic.es/tools/venny/). Heatmaps of gene expression correlations were designed with Plotly (https://plot.ly/).
